# Coagulation dysfunction events associated with tigecycline: a real-world study from FDA adverse event reporting system (FAERS) database

**DOI:** 10.1186/s12959-022-00369-z

**Published:** 2022-03-05

**Authors:** Mingxing Guo, Jinwei Liang, Dandan Li, Ying Zhao, Wanyi Xu, Lei Wang, Xiangli Cui

**Affiliations:** 1 Department of Pharmacy, Beijing Friendship Hospital, Capital Medical University, Beijing, China; 2 School of Automation Science and Electrical Engineering, Beihang University, Beijing, China

**Keywords:** Tigecycline, Coagulation dysfunction, FDA adverse event reporting system

## Abstract

**Background:**

Tigecycline has broad-spectrum anti-bacterial activity and often used for critically ill patients with complicated infections. Only a few clinical studies have reported the coagulation disorder induced by tigecycline. The aim of this study was to investigate the association between tigecycline and coagulation dysfunction using the US Food and Drug Administration Adverse Event Reporting System (FAERS) database.

**Method:**

Data from January 2005 to December 2020 in FAERS were retrieved. We investigated the clinical characteristics of the coagulation dysfunction events and conducted disproportionality analysis by using reporting odds ratios (ROR) to compare tigecycline with the full database and other antibiotics.

**Results:**

The total number of reports of coagulation dysfunction related to tigecycline as the primary suspect drug was 223. The median time to event of the coagulation dysfunction events was 10 (interquartile range [IQR] 6.75–13) days. 80.72% coagulation-related adverse events appeared within the first 14 days since the initiation of tigecycline administration. The overall ROR (95% CI) for coagulation-related adverse events was 3.55 (3.08, 4.09). The RORs (95% CI) for thrombocytopenia, hypofibrinogenaemia, coagulopathy, activated partial thromboplastin time prolonged, international normalized ratio increased, prothrombin time prolonged were 8.21 (6.34, 10.62), 705.41 (526.81, 944.54), 30.67 (21.92, 42.92), 42.98 (24.85, 74.31), 4.67 (2.51, 8.71), and 27.99 (15.01, 52.19), respectively. In analyses stratified on comparing tigecycline to vancomycin and daptomycin, significant coagulation dysfunction signals were found with the RORs (95% CI) 2.74 (2.34, 3.22) and 3.08 (2.57, 3.70).

**Conclusions:**

We found a strong signal of high frequency of reporting coagulation dysfunction in tigecycline. Health professionals should be aware of the potential coagulation disorders risk and monitor coagulation parameters during anti-bacterial therapy with tigecycline, particularly the need to monitor fibrinogen levels.

**Supplementary Information:**

The online version contains supplementary material available at 10.1186/s12959-022-00369-z.

## Introduction

Tigecycline is a glycylcycline class antibacterial drug which exerts antibacterial effects by inhibiting bacterial protein synthesis, especially against multidrug-resistant (MDR) bacteria such as methicillin-resistant *Staphylococcus aureus* (MRSA), vancomycin-resistant *Enterococcus* (VRE), extended-spectrum β-lactamase (ESBL) - producing *Enterobacteriaceae*, carbapenem-resistant *Enterobacteriaceae*, and MDR *Acinetobacter* spp. [1]. It is increasingly used in difficult-to-treat infections that do not respond to first-line antibiotics [2]. Tigecycline does not require dose adjustment in renal impairment and has minimal drug interactions, which make it suitable for patients with severe clinical infections.

Tigecycline is well tolerated. In clinical trials and post-marketing experience, the safety summary of the use of tigecycline shows that nausea and vomiting are the most common adverse reactions [3]. However, some case reports indicate that tigecycline seems to induce coagulation dysfunction, manifested by bleeding and abnormal coagulation parameters [4–6]. To our knowledge, only a few clinical studies have reported the adverse event of coagulation dysfunction induced by tigecycline [7, 8].

FDA Adverse Event Reporting System (FAERS) is a database designed to support the FDA’s post-marketing monitoring program for drugs and therapeutic biological products. The database includes all adverse drug event (ADE) information and medication error information collected by the FDA. We conducted this study to examine the association between tigecycline and coagulation dysfunction, and compared ROR of coagulation dysfunction caused by tigecycline and other antibiotics. All data analysis are based on FAERS database.

## Methods

### Study design and data sources

The interventions of interest was tigecycline. All records in the FAERS database (https://open.fda.gov/data/faers/) from January 2005 to December 2020 were included in this study. We used both generic names and brand names to identify tigecycline and control drugs (Appendix Table 1).

Adverse events (AE) were included when they were considered the “Primary Suspect” drug (those drugs directly suspected of causing the adverse events when submitted in the case report). To validate the robustness of the findings, we conducted four specific comparisons: (1) compared tigecycline with the full database; (2) compared tigecycline with the drugs for treatment of methicillin-resistant *Staphylococcus aureus* (MRSA): vancomycin, linezolid and daptomycin; (3) compared tigecycline with the drugs for treatment of extended-spectrum β-lactamase (ESBL)-producing *Enterobacteriaceae:* meropenem and imipenem-cilastatin; (4) compared tigecycline with the drugs with *N*-methyltetrazolium side chain: cefoperazone and other similar drugs.

We identified coagulation dysfunction events which were coded in preferred terms (PT) using Medical Dictionary for Regulatory Activities terms (V22.0) (Appendix Table 2). The primary outcome was overall coagulation-related adverse events. The secondary outcomes were the high frequency reporting of coagulation dysfunction events: thrombocytopenia, hypofibrinogenaemia, coagulopathy, activated partial thromboplastin time prolonged, international normalized ratio increased, prothrombin time prolonged. All the data extraction were performed by Python. Python is a programming language that can retrieve ADR and drug related information through programming algorithms.

### Statistical analysis

In pharmacovigilance study, disproportionality emerges when a specific adverse event is associated with a given drug. We performed disproportionality analyses using the reporting odds ratio (ROR), which is a statistical aid of signal detection to calculate the proportions of specified reported reactions for a given drug [9]. The ROR was calculated by dividing the odds of coagulation dysfunction events reporting for the drug of interest by the odds of coagulation dysfunction events reporting for the comparison drug. The odds of coagulation dysfunction events reporting for a drug were calculated by dividing the number of drug case reports listing“coagulation dysfunction events” by the number of drug case reports listing all other adverse events. In this study, tigecycline was the drug of interest. A signal of increased coagulation dysfunction risk was defined as a ROR ≥ 2.0 with the number of cases ≥3 [9, 10].

To assess the relationship between the control group itself and abnormal coagulation events, subgroup analyses were performed for the secondary outcomes of the high frequency reporting of coagulation dysfunction events in the different comparisons.

## Results

### Descriptive analysis

Overall, from January 2005 to December 2020, 1517 reports listing tigecycline were identified as the primary suspect drug. The number of abnormal coagulation function events was 223 (14.70%) for tigecycline. The clinical characteristics of patients with tigecycline induced abnormal coagulation function were described in Table 1. The coagulation disorders reporting associated with tigecycline were slightly higher in male than in female (56.05% vs. 30.94%, *P* < 0.05), and 43.95% of them are elderly patients (age ≥ 65 years old). Healthcare professionals submitted cases higher than non-health-care workers (58.30% vs. 37.22%, *P* < 0.05). The number of reported cases of tigecycline-related abnormal coagulation events was increasing year by year. And from 2016 to 2020, a total of 101 cases were reported, accounting for 45.29%. The case-fatality rate of the reported coagulation disorders was 20.18%. From the collected report results, there was no increase in coagulation abnormalities between the loading dose of tigecycline and the non-loading dose (*P* = 0.41). Moreover, the use of standard maintenance doses (100 mg/day) of tigecycline reported higher coagulation events than the high dose maintenance group (200 mg/day or higher) with the number of reported cases was 111:34, but not significant (*P* = 0.43)”.

**Table 1:** Clinical characteristics of patients with tigecycline-associated coagulation dysfunction events

	No. of coagulation-dyfunction in tigecycline (%)	No. of other AEs in tigecycline (%)
**Gender**		
Male	125 (56.05)	520 (40.19)
Female	69 (30.94)	501 (38.72)
Missing	29 (13.00)	273 (21.10)
**Age**		
< 65	69 (30.94)	470 (36.32)
≥ 65	98 (43.95)	360 (27.82)
Missing	56 (25.11)	464 (35.86)
**Reporter**		
Healthcare workers	130 (58.30)	787 (60.82)
Non-health care workers	83 (37.22)	457 (35.32)
Unknown	10 (4.48)	50 (3.86)
**Year**		
2005–2010	51 (22.87)	420 (32.46)
2011–2015	71 (31.84)	418 (32.30)
2016–2020	101 (45.29)	456 (35.24)
**Outcome**		
Death	45 (20.18)	340 (26.28)
Life-threatening	28 (12.56)	61 (4.71)
Disability	0 (0.00)	16 (1.24)
Hospitalization	35 (15.70)	308 (23.80)
Other serious	107 (47.98)	405 (31.30)
Required intervention	1 (0.45)	4 (0.31)
Missing	7 (3.14)	160 (12.36)
**Dosing regimen**		
Loading dose	28 (12.56)	86 (6.64)
Non-loading dose	121 (54.26)	305 (23.57)
Standard maintenance dose^a^	111 (49.78)	481 (37.17)
High maintenance dose^c^	34 (15.25)	124 (9.58)
**Time to event**	10 (IQR 6.75–13)	
**Total**	**223**	**1294**

^a^ Standard maintenance dose means the dose of tigecycline administered at 100 mg/d

^b^ High maintenance dose means the dose of tigecycline administered at 200 mg/d or higher

**Fig. 1:**
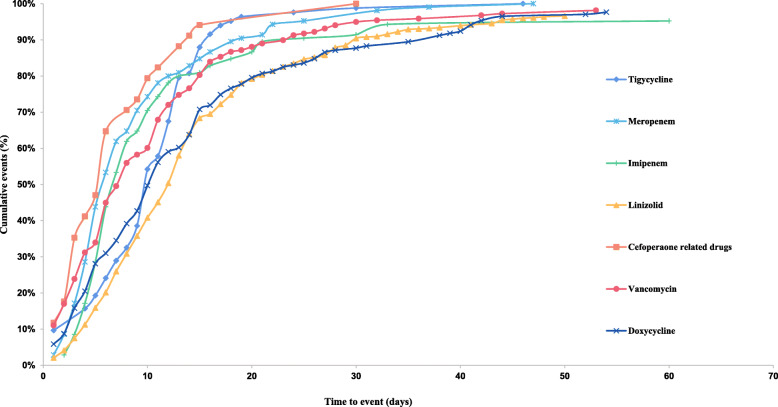
Cumulative event (%) of coagulation events since the initiation of tigecycline and the control drugs

The median time to event of the coagulation dysfunction of tigecycline use were 10 (interquartile range [IQR] 6.75–13) days. 80.72% coagulation-related adverse events appeared within the first 14 days since the initiation of tigecycline administration (Fig. 1).

### Signal values associated with overall coagulation-related adverse events

**Fig. 2:**
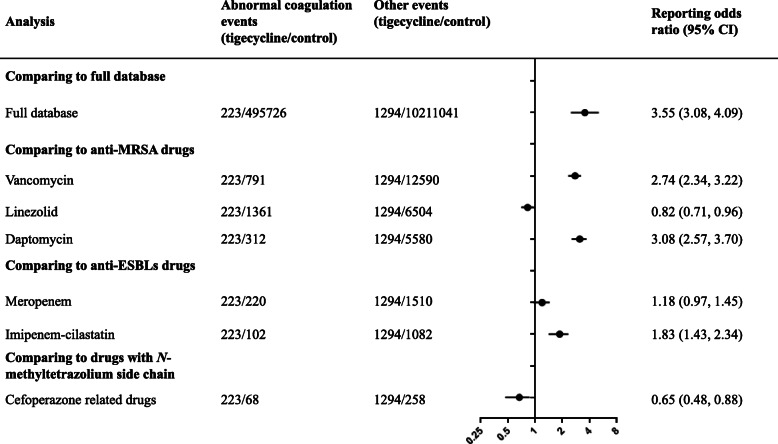
Overall coagulation dysfunction signals and RORs comparing tigecycline with the full database and other antibiotics

For the three specific comparisons, there was a strong signal for increased coagulation dysfunction risk (ROR 3.55, 95% CI: 3.08, 4.09), when comparing tigecycline with the full database (Fig. 2). Compared with anti-MRSA drugs, tigecycline significantly increased the risk of coagulation dysfunction compared with vancomycin and daptomycin, with the RORs (95% CI) 2.74 (2.34, 3.22) and 3.08 (2.57, 3.70). However, the signal was not found in the comparison with linezolid with the ROR (95% CI) 0.82 (0.71, 0.96). Analysis showed that tigecycline was not associated with a higher risk of abnormal coagulation function compared with anti-ESBLs drugs of meropenem and imipenem-cilastatin and drugs with *N*-methyltetrazolium side chain: cefoperazone related drugs (ROR 1.18 [0.97, 1.45], 1.83 [1.43, 2.34], 0.65 [0.48, 0.88], respectively).

### Signal values associated with different coagulation dysfunction events

**Fig. 3:**
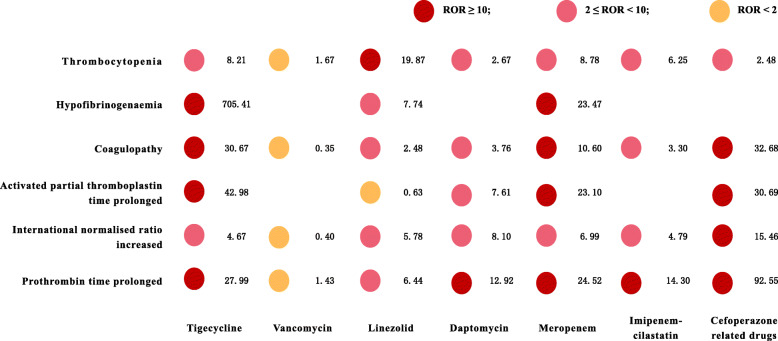
Subgroup analysis of different coagulation dysfunction events in tigecycline and other antibiotics

In the secondary outcomes for the high frequency reporting of different coagulation dysfunction events associated with tigecycline, the RORs (95% CI) for thrombocytopenia, hypofibrinogenaemia, coagulopathy, activated partial thromboplastin time prolonged, international normalized ratio increased, and prothrombin time prolonged were 8.21 (6.34, 10.62), 705.41 (526.81, 944.54), 30.67 (21.92, 42.92), 42.98 (24.85, 74.31), 4.67 (2.51, 8.71), and 27.99 (15.01, 52.19), respectively (Fig. 3). Notably, a very strong signal of higher frequency of reporting hypofibrinogenaemia associated with tigecycline was found.

## Discussion

Overall, we observed a strong signal of higher frequency of reporting coagulation dysfunction events associated with tigecycline compared with all the other drugs. Among the different coagulation dysfunction events, thrombocytopenia, hypofibrinogenaemia, coagulopathy, activated partial thromboplastin time prolonged, international normalized ratio increased, and prothrombin time prolonged were the high frequency reporting events and have strong correlation signals. Hypofibrinogenaemia had an outstanding contribution to the reporting of coagulation disorders of tigecycline with the ROR (95% CI) 705.41 (526.81, 944.54).

Tigecycline has increasingly been used due to the higher incidence of multidrug-resistant bacteria induced infections. Post-marketing data signaling increased mortality rates in tigecycline treated patients have brought its use in patients with complicated infections into question, prompting many practitioners to consider other potential adverse effects not found in initial studies. In our study, the number of reported cases of tigecycline related abnormal coagulation events was increasing year by year and were mainly submitted by healthcare professionals, but the number of reported cases of other adverse events in tigecycline did not increase as showed in Table 1. It shows that tigecycline related coagulation disorder has been paid more and more attention.

A literature review reported that tigecycline induced coagulopathy usually manifests as the dose dependent prolongation of prothrombin time and activated partial thromboplastin time and a reduction in the fibrinogen level [11]. However, in our study, most patients were given the recommended dose (100 mg/day). And regardless of whether a loading dose is given or not, abnormal coagulation events may occur in patients given conventional doses. We found a strong signal of hypofibrinogenaemia associated with tigecycline use. A retrospective analysis of the use of tigecycline to treat severe infections also found that a recommended dose of tigecycline can result in a reduction in plasma fibrinogen levels, which returned to normal after the cessation of treatment [12]. And they recommended regular monitoring of coagulation during tigecycline treatment.

Some studies have found that duration of tigecycline treatment > 14 days was a independent variable associated with hypofibrinogenaemia [8, 13]. In our study, 80.72% of patients developed coagulation dysfunction in 2 weeks of tigecycline use. The median time to event of the coagulation dysfunction events was 10 (IQR 6.75–13) days. A retrospective case control study was consistent with our findings which showed that hypofibrinogenaemia developed at a median of 6 (4–8) days after tigecycline treatment [14]. We suggest health professionals be aware of the potentially risk of tigecycline-associated hypofbrinogenemia and monitor coagulation function during treatment, although the first 2 weeks of tigecycline use.

Patients using tigecycline may have abnormal coagulation dysfunction, and at the same time, patients with drug-resistant strains may use multiple antimicrobial combination treatment strategies. For example, for *acinetobacter baumannii* infection, combined antibiotic treatment is a common strategies [15]. In addition, tigecycline may not reach sufficient levels in the serum, urinary tract, or central nervous system to successfully treat infections in these conditions, and there may be need combination medications. For such patients, the effect of tigecycline on coagulation dysfunction may be exaggerated. Therefore, a subgroup analysis was used to compare tigecycline to the different antibiotics. In analyses stratified on comparing tigecycline to vancomycin and daptomycin, a increased coagulation dysfunction event reporting were found (ROR > 2). Several studies have reported that daptomycin has a dose-dependent effect on the artificial prolongation of prothrombin time and prolongation of international normalized ratio [16, 17]. When comparing tigecycline with imipenem-cilastatin, there was a moderate signal of higher frequency of reporting coagulation dysfunction events with ROR (95% CI) 1.83 (1.43, 2.34). A randomized open-label study found that compared to imipenem-cilastatin, tigecycline was associated with a significant decrease in fibrinogen levels, following cytoreductive surgery and hyperthermic intraperitoneal chemotherapy [18].

In analyses stratified on comparing to linezolid, meropenem, and cefoperazone related drugs as the control groups, no signal of increased coagulation dysfunction event reporting were found (ROR < 2). The results might be related to the influence of the control drug itself on coagulation function. It has been reported that linezolid can also cause thrombocytopenia [19–21]. Some case reports has prompted that meropenem had the risk of thrombocytopenia and pancytopenia [22, 23]. Cefoperazone has an *N*-methyltetrazole side chain at the 3-position of the cephalosporin nucleus and thus possesses the potential for producing hypoprothrombinaemic bleeding [24–26]. This further explained why tigecycline had no obvious coagulation abnormal signal compared with these antibacterial drugs.

In our center, we observed one case of low levels of fibrinogen associated with the use of tigecycline, but the mechanism remained unclear [27]. Since we were unable to analyze the effect of the patient’s disease status on coagulation in this study, we cannot claim these data show that tigecycline causes coagulation dysfunction. Fibrinogen decreases, combined with prolongation of clotting time or/and platelet decreases, can be indicative of coagulopathy and increase the risk of major bleeding, prolonged hospitalization, and mortality. In this regard, fibrinogen levels may be a biomarker of tigecycline related coagulopathy. Other related factors that can affect the metabolism of fibrinogen include liver dysfunction, active bleeding, fibrinogen degradation accelerated by acidosis, and fibrinogen inhibition caused by low body temperature. The mechanism for the fibrinogen decrease found with tigecycline is unclear. Some scholars have pointed out that tigecycline can inhibit IL-6 expression in leucocytes [28]. Since IL-6 increases fibrinogen blood levels by stimulating gene expression, tigecycline might decrease fibrinogen plasma levels by inhibiting IL-6 synthesis [29].

To our knowledge, we reported the first and most comprehensive analysis of the risk of coagulation dysfunction associated with tigecycline from the real-world database. We found a strong signal of high frequency of reporting coagulation dysfunction in tigecycline. Further we found that hypofibrinogenaemia had an outstanding contribution to the reporting of coagulation disorders of tigecycline. Certainly, there are inherent limitations with disproportionality analysis methods based on FAERS data. First, FAERS data is prone to reporting biases and missing data, and we were unable to fully control for confounding. Second, because the data lacks a meaningful denominator and cannot exclude prevalent case, we cannot estimate true risk or assess the incidence rate. Lastly, due to lack of the patient’s underlying disease status, we did not consider the effect of disease factor which could be an important factor in coagulation abnormal events.

## Conclusions

We found a strong signal of high frequency of reporting coagulation dysfunction in tigecycline. The median time to event of the coagulation dysfunction events were 10 days. Most coagulation-related adverse events appeared within the first 14 days since the initiation of tigecycline administration. Moreover, we observed strong signals of higher frequency of reporting coagulation dysfunction events associated with tigecycline in the events of thrombocytopenia, hypofibrinogenaemia, coagulopathy, activated partial thromboplastin time prolonged, international normalised ratio increased, and prothrombin time prolonged. Hypofibrinogenaemia had an remarkable contribution to the reporting of coagulation disorders of tigecycline. Health professionals should be aware of the potential coagulation disorders risk and monitor coagulation parameters during tigecycline therapy, particularly fibrinogen levels.

## Supplementary Information

**Additional file 1: Appendix Table 1.** Search terms for tigecycline and its control drugs.

**Additional file 2: Appendix Table 2.** Search terms for adverse events related to coagulation dysfunction.

## Data Availability

The data used during the current study are available from FAERS database (https://open.fda.gov/data/faers/).
