# Supplementary material for: Coagulation dysfunction events associated with tigecycline: a real-world study from FDA adverse event reporting system (FAERS) database
Source: Thromb J. 2022 Mar 5;20:12. doi: 10.1186/s12959-022-00369-z (PMC8898466; doi:10.1186/s12959-022-00369-z)
Supplement: Supplementary file 1 — Additional file 1: Appendix Table 1. Search terms for tigecycline and its control drugs. [file 12959_2022_369_MOESM1_ESM.docx]

**Appendix Table 1.** Search terms for tigecycline and its control drugs.

| **Search object** | | **Search term** |
| --- | --- | --- |
| Target drug | Tigecycline | Tigecycline, tigecycline-hydrochloride, Tygacil |
| Control drugs | Linezolid | Linezolid, Zyvox |
|  | Daptomycin | Daptomycin, Cubicin |
|  | Vancomycin | Vancomycin, Vancomycin hydrochloride, First Vancomycin Rx, Firvanq KIT, Vancocin hydrochloride, Vancoled, Vancomycin HCL, Vancor |
|  | Meropenem | Meropenem, Meropenem anhyarous, Meropenem/sodium chloride, Merrem, Vabomere |
|  | Imipenem and cilastatin | Imipenem and cilastatin, Imipenem and cilastatin sodium, cilastatin sodium/Imipenem, cilastatin/cilastatin sodium/Imipenem, cilastatin/Imipenem anhydrous/relebactam anhydrous, Primaxin, Recarbrio |
|  | Cefoperazone related drugs | Cefoperazone, Cefoperazone sodium, Cefoperazone sodium/sulbactam, Cefoperazone/sulbactam, Cefoperazone/sulbactam sodium, Cefoperazone/tazobactam sodium, Cefobid, Sulperazon |
