# Supplementary material for: Coagulation dysfunction events associated with tigecycline: a real-world study from FDA adverse event reporting system (FAERS) database
Source: Thromb J. 2022 Mar 5;20:12. doi: 10.1186/s12959-022-00369-z (PMC8898466; doi:10.1186/s12959-022-00369-z)
Supplement: Supplementary file 2 — Additional file 2: Appendix Table 2. Search terms for adverse events related to coagulation dysfunction. [file 12959_2022_369_MOESM2_ESM.docx]

**Appendix Table 2.** Search terms for adverse events related to coagulation dysfunction.

| **Search object** | **Search term** |
| --- | --- |
| Coagulation abnormalities | Hypofibrinogenaemia, Coagulopathy, Hyperfibrinolysis, Thrombocytopenia, Platelet count decreased, Platelet count abnormal, Activated partial thromboplastin time prolonged, Activated partial thromboplastin time shortened, Activated partial thromboplastin time ratio decreased, Activated partial thromboplastin time abnormal, Activated partial thromboplastin time ratio abnormal, Activated partial thromboplastin time ratio fluctuation, Activated partial thromboplastin time ratio increased, Prothrombin time prolonged, Prothrombin time ratio increased, Prothrombin time ratio decreased, Prothrombin time ratio abnormal, Prothrombin time abnormal, Haemorrhage, bleeding, Coagulation time prolonged, Coagulation time abnormal, Microangiopathic haemolytic anaemia, Haemorrhagic, Disseminated intravascular coagulation, Thrombocytopenic purpura, International normalised ratio fluctuation, International normalised ratio increased, International normalised ratio decreased, International normalised ratio abnormal, Fibrin decreased, Blood fibrinogen decreased, Blood fibrinogen increased, Blood fibrinogen abnormal, Fibrinolysis increased, Fibrinolysis abnormal, Fibrin degradation products increased, Fibrin D dimer increased, Fibrin D dimer decreased, Fibrin abnormal, Acquired dysfibrinogenaemia, Hypocoagulable state, Antithrombin III decreased, Thrombin-antithrombin III complex decreased, Acquired antithrombin III deficiency, Antithrombin III abnormal, Antithrombin III deficiency, Antithrombin III increased, Thrombin-antithrombin III complex abnorma, Thrombin-antithrombin III complex increased, Coagulation factor decreased, Coagulation factor deficiency, Coagulation factor IX level abnormal, Coagulation factor IX level decreased, Coagulation factor V level abnormal, Coagulation factor V level decreased, Coagulation factor VII level abnormal, Coagulation factor VII level decreased, Coagulation factor VIII level abnormal, Coagulation factor VIII level decreased, Coagulation factor X level abnormal, Coagulation factor X level decreased, Coagulation factor XI level abnormal, Coagulation factor XI level decreased, Coagulation factor XII level abnormal, Coagulation factor XII level decreased, Coagulation factor XIII level abnormal, Coagulation factor XIII level decreased |
